# Low fraction of the 222K PrP variant in the protease-resistant moiety of PrP^res^ in heterozygous scrapie positive goats

**DOI:** 10.1099/jgv.0.000843

**Published:** 2017-07-13

**Authors:** Maria Mazza, Chiara Guglielmetti, Francesco Ingravalle, Sonia Brusadore, Jan P. M. Langeveld, Loukia V. Ekateriniadou, Olivier Andréoletti, Cristina Casalone, Pier Luigi Acutis

**Affiliations:** ^1^​ Italian Reference Centre for TSEs, Istituto Zooprofilattico Sperimentale del Piemonte, Liguria e Valle d'Aosta, Turin, Italy; ^2^​ Biostatistic, Epidemiology and Risk Analysis Unit, Istituto Zooprofilattico Sperimentale del Piemonte, Liguria e Valle d'Aosta, Turin, Italy; ^3^​ Wageningen Bioveterinary Institute, Lelystad, The Netherlands; ^4^​ National Agricultural Research Foundation, Veterinary Research Institute, Thessaloniki, Greece; ^5^​ UMR INRA ENVT 1225, Interactions Hôtes Agents Pathogènes, Ecole Nationale Vétérinaire de Toulouse, Toulouse, France

**Keywords:** 222K, goat, prion protein, scrapie, Western blot, PrP, heterozygous

## Abstract

The presence of lysine (K) at codon 222 has been associated with resistance to classical scrapie in goats, but few scrapie cases have been identified in 222Q/K animals. To investigate the contribution of the 222K variant to PrP^res^ formation in natural and experimental Q/K scrapie cases, we applied an immunoblotting method based on the use of two different monoclonal antibodies, F99/97.6.1 and SAF84, chosen for their different affinities to 222K and 222Q PrP variants. Our finding that PrP^res^ seems to be formed nearly totally by the 222Q variant provides evidence that the 222K PrP variant confers resistance to conversion to PrP^res^ formation and reinforces the view that this mutation has a protective role against classical scrapie in goats.

Scrapie, an infectious neurodegenerative disease within the group of transmissible spongiform encefalopathies (TSEs), also known as prion diseases, affects sheep and goats. Like all TSEs, the pathological process occurs as a consequence of the conversion and accumulation in the central nervous system of an abnormal isoform (PrP^res^) of the normal cellular prion protein (PrP^C^).

Susceptibility to classical scrapie in sheep is influenced by the different polymorphisms of the PrP encoding gene at position 136 [valine (V) and alanine (A)], 154 [arginine (R) and histidine (H)], and the four variants at codon 171 [glutamine (Q), R, H and lysine (K)]. The VRQ and ARQ alleles are associated with high susceptibility, whereas the ARR allele confers a high level of resistance in both natural and experimental conditions [1, 2]. To control classical scrapie in sheep populations, European Union Member States have established breeding programmes based on the selection of the resistant ARR allele. Also in goats, field and experimental studies evidenced associations between certain polymorphisms of the *PRNP* gene and susceptibility/resistance to classical scrapie [3].

Among them, the polymorphism at codon 222 (Q222K) yielded the most promising results. In particular, the presence of lysine (K) at codon 222 has been shown to be associated with resistance to classical scrapie and bovine spongiform encephalopathy (BSE) agents in several field and experimental studies [4–10]. The protective role of the 222K allele in goats, observed also in heterozygous 222 animals (222Q/K), makes this allele a good candidate for breeding programmes to control and eradicate scrapie in goat herds. However, few natural scrapie cases have been identified in 222Q/K goats in Greece and France, and intracerebral experimental transmission was successful after long incubation periods and with low attack rates in both heterozygous (222Q/K) and homozygous (222K/K) goats [11–13]. Although carrying this polymorphism does not fully protect against classical scrapie, the low incidence of natural scrapie in goats carrying these PrP variants suggests a low tendency of PrP with lysine at position 222 to convert into the pathological isoform (PrP^res^). While a few scrapie positive homozygous ARR carriers among sheep have been reported [14, 15], *in vitro* conversion studies and prion allotype studies in naturally scrapie infected ARR/VRQ carriers showed a low tendency of ovine PrP 171R variant to become PK resistant [16–18]. In light of these findings, a differential contribution by the PrP 222Q and 222K variants to pathological prion protein (PrP^res^) formation could be hypothesized in goats as well, and this merits verification.

In a previous study, we demonstrated, using a Western blot method, a total inhibition by lysine at position 222 on the binding of the monoclonal antibody (mAb) F99/97.6.1 to goat PrP^C^ in healthy goat brain and we proposed the ratio between the signal intensity given by the mAbs F99/97.6.1 and SAF84 (referred to simply as F99/SAF84) to discriminate goats carrying the 222Q/Q, 222Q/K and 222K/K PrP genotypes [19]. In the present study, we applied the same approach to investigate the contribution of the PrP 222K variant to PrP^res^ formation in natural and experimental scrapie positive goats.

For this purpose, brain tissue samples from Greek, French and Italian goats were derived from animals with a different amino acid combination at position 222 of the prion protein and diagnosed as either negative or positive for scrapie (Table 1). The tissue samples were kept frozen until analysis.

**Table 1.** Positive and negative goat samples analysed in this study

Sample no.	Sample ID	PrP 222	Scrapie
1	Gr 195	Q/K	−
2	Gr 247	Q/K	−
3	It 19 434	Q/K	−
4	It 17 778	Q/K	−
5	It 19 564	Q/K	−
6	It 19 431	Q/K	−
7	Fr 60 120	Q/K*	−
8	Fr 60 101	Q/K*	−
9	It 1	Q/Q	−
10	It 2	Q/Q	−
11	It 3	Q/Q	−
12	It 4	Q/Q	−
13	Gr 05	Q/K	+
14	Fr 60 121	Q/K*	+
15	Fr 60 135	Q/K*	+
16	Gr 91	Q/Q	+
17	Gr 55	Q/Q	+
18	Gr 18	Q/Q	+
19	It 54 817	Q/Q	+
20	It 72 366	Q/Q	+
21	It 77 811	Q/Q	+
22	It 7395	Q/Q	+
23	Fr 70 115	K/K*	−
24	Fr 70 505	K/K*	+
25	Fr 60 572	K/K*	+
26	Fr 60576	K/K*	+

Gr, Fr, It: Greek, French, and Italian goats; −, +: negative or positive scrapie cases.

*Experimental i.c. scrapie cases.

Prion protein extraction (PrP^C^ and PrP^res^) from each sample was carried out as previously reported [19]. 10 % (w/v) homogenates of nervous tissue were prepared in lysis buffer [10 % N-lauroylsarcosine diluted in Tris-buffered saline (TBS), pH 7.4] and clarified by centrifugation at 22,000 RCF for 20 min. A volume of 10 µl of a 100 mM phenylmethylsulphonyl fluoride solution was added to 1 ml of each supernatant from negative scrapie goats; equal aliquots obtained from positive scrapie samples were digested by proteinase K (PK, 40 µg ml^−1^) at 37 °C for 1 h. The samples were then centrifuged at 215 000 RCF for 1 h; the pellets were dissolved in Laemmli buffer and subjected to sodium dodecyl sulfate-polyacrylamide gel electrophoresis on a 12 % handmade mini-gel and then transferred onto polyvinylidene difluoride membranes. PrP immunodetection was performed in parallel using mAbs F99/97.6.1 (4 µg ml^−1^) and SAF84 (1 µg ml^−1^), which recognize PrP residues 220–225 and 163–173, respectively, and show equal chemiluminescence signals at the concentrations used. Antibody binding was detected with an alkaline phosphatase-conjugated goat anti-mouse immunoglobulin G (0.1 µg ml^−1^) (Thermo Fisher Scientific, Invitrogen, *catalogue* 62–6822) and immuno-reactivity was visualized by a chemiluminescent reaction with Novex® AP Chemiluminescent Substrate CDP-Star® (ThermoFisher Scientific, Invitrogen, *catalogue* WP20002). Each sample was analysed at least in triplicate, in different runs, and the images of the blots were captured with an image analyser (Uvitech, Cambridge, UK). The data were then analysed using commercial software (UVI Prochemi software, Uvitech, Cambridge, UK).

The digital data were used to calculate the ratio between F99/97.6.1 and SAF84 signals.

A non-parametric approach was applied to evaluate the presence of significant differences in the ratios among the four different groups of samples (Q/K positive, Q/K negative, Q/Q positive, Q/Q negative). The Kruskal–Wallis test [20] was applied to the median value of the replicates measured for each goat. Pairwise comparisons between each couple of groups were performed using the Mann–Whitney two-sample statistic [21, 22]. K/K samples were excluded from the analysis since the ratios were always zero.

Subsequent to statistical analysis of the ratios, the amounts of PrP^res^ 222Q and 222K present in positive scrapie 222Q/K goats were calculated according to Jacobs *et al.* [18]: the fraction of the PrP 222Q product was obtained by applying the formula ratio*_x_*/ratio*_Q/Q_*_pos_, where ratio*_x_* is the F99/SAF84 ratio of an unknown sample and ratio*_Q/Q_*_pos_ is the F99/SAF84 ratio determined for Q/Q homozygous positive material, which was an average measurement of at least three different 222Q/Q samples applied in the same run; likewise, the fraction of the PrP 222K product was deduced according to the formula (ratio*_Q/Q_*_pos_ – ratio*_x_*)/ratio*_Q/Q_*_pos_ and then reported as a percentage value.

Western blot analysis with mAb F99/97.6.1 revealed no PrP signal in any of the 222K/K goat samples, either negative (PrP^C^, no PK digestion applied) or positive, natural or experimental (PK digested PrP^res^), whereas analysis with mAb SAF84 detected PrP^C^ and PrP^res^ in all samples. In all positive scrapie samples (natural and experimental isolates), PrP^res^ signals showed a typical electrophoretic pattern of classical scrapie characterized by three bands corresponding to the di-, mono-, and unglycosylated forms migrating at approximately 30, 25 and 20 kDa, respectively (Fig. 1, panels 1 and 2). In the negative scrapie goats, analysis with both monoclonal antibodies showed PrP^C^ signals represented by a single protein with a molecular weight of approximately 25 kDa (Fig. 1, panel 3), as observed in a previous study [19].

**Fig. 1.**
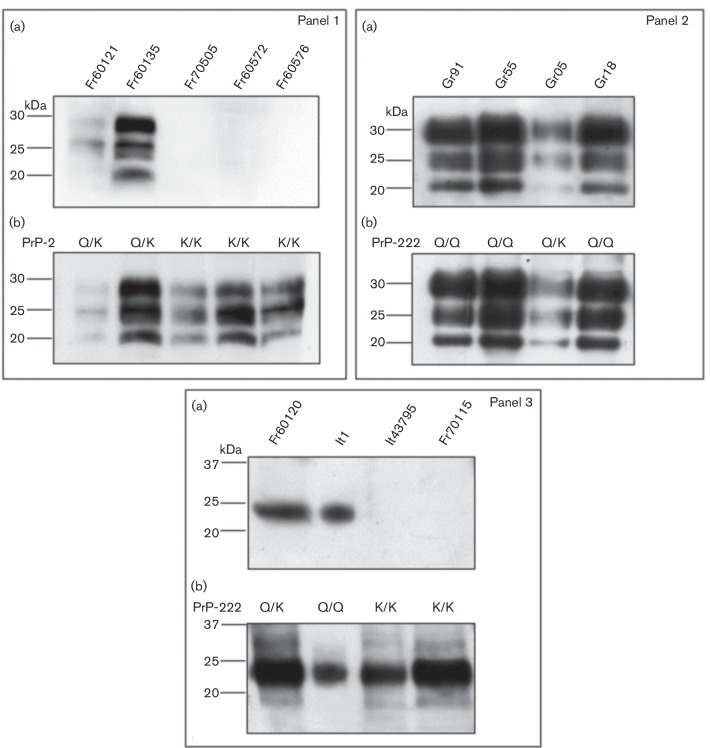
Western blot analysis of proteinase K-treated PrP^res^ extracted from the brain tissue of French (panel 1; experimental scrapie isolates), Greek and Italian goats (panel 2; natural scrapie cases) and no-pK treated PrP^C^ (panel 3; negative samples) with different polymorphisms at PrP codon 222 (222Q/Q,222Q/K and 222 K/K). PrP^res^ and PrP^C^ were detected in parallel by F99/97.6.1 (a) and SAF84 (b).

The mean F99/SAF84 ratio (±standard deviation) was calculated for each sample (Fig. 2 and Table S1, available in the online Supplementary Material).

**Fig. 2.**
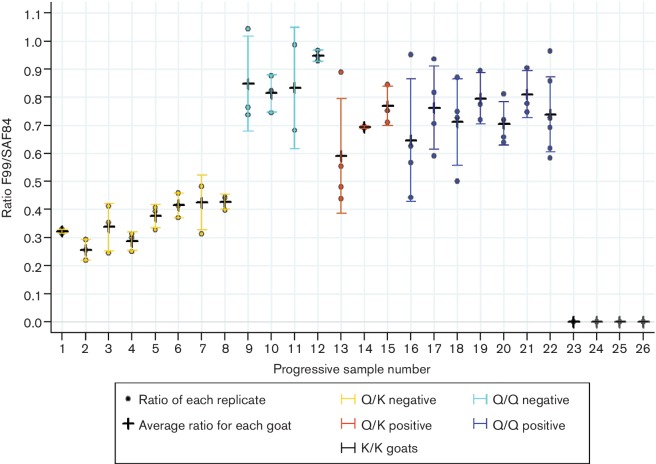
Scatter plot of the F99/SAF84 ratio obtained from Greek, French and Italian goat samples showing different genotypes at *PRNP* gene codon 222 (222Q/Q, 222Q/K and 222 K/K). Crosses indicate the average F99/SAF84 ratio for each sample with standard deviation (vertical lines).

The ratio of the optical densities revealed that, overall, the reactivity was similar for the positive 222Q/K goats and the 222Q/Q samples, except for the natural 222Q/K goat scrapie sample Gr 05, in which the ratio values were slightly lower than in the experimental goat scrapie samples (Fr 60121 and Fr 60135). Statistical analysis showed no difference between the ratios of the Q/K positive samples and those of the Q/Q positive samples.

Taken together, these data show that PrP^res^ seems to be composed mainly of the 222Q variant in the 222Q/K positive scrapie goats. The amount of the 222K variant calculated according to the above-described formula showed that the fraction of PrP^res^ composed of the 222K was 0 and 5.5 %, respectively, in the experimental positive French cases (Fr 60 121 and Fr 60 135), whereas it was 19 % in the Greek positive natural case (Gr 05).

Contrary to the equimolar amounts expected from a heterozygous animal, our results show that a low fraction of the 222K protein in PrP^res^ was present in the Q/K positive goats. Similar features for the resistant ARR allele were found by studies investigating the PrP^res^ composition of scrapie positive ARR/VRQ sheep. Using an approach similar to ours, Jacobs [18] estimated a level of 0–9 % of the ARR allele. In another study based on matrix-assisted laser desorption and ionization time-of-flight (MALDI-TOF) mass spectrometry analysis, no contribution from the ARR variant to PrP^res^ formation emerged in the ARR/VRQ sheep, while both variants were equally represented in the normal isoform [23].

The reduced contribution by the 222K allele to PrP^res^ formation in the heterozygous scrapie goats showed a poor tendency of the 222K prion protein to convert from the normal into the pathological isoform. This observation is shared by Eiden *et al.* [24], who found no conversion of the 222K PrP variant in a cell-free assay. It has been hypothesized that the insertion of an additional positive charge, given by the lysine at position 222 of the caprine PrP, is assumed to interfere with PrP^C^-PrP^res^ interaction, resulting in a null or low conversion rate into PrP^res^ [13, 25, 26]. Moreover, interference by the susceptible 222Q allele on 222K PrP conversion into PrP^res^ in heterozygosis could also be hypothesized. This effect was described in previous studies based on *in vitro* conversion assays in sheep and goat PrP [17, 24] and could also explain the fact that 222K PrP converted in experimental challenge homozygous K/K animals after a long incubation period.

Our study revealed a higher contribution by the 222K variant to PrP^res^ formation in the Greek goat affected by natural scrapie as compared to the French experimental cases. This major conversion of the 222K variant into a pathological isoform during natural infection could be due to: (i) the route of infection different from the experimental cases; (ii) a particularly high infectious dose; (iii) the properties of the scrapie strain involved. The incubation period could be ruled out, given that it was similar between the experimental Q/K cases and the age of disease onset for natural cases (≅6 years). The available data do not allow confirmation of any of the hypotheses above, but the epidemiological and molecular typing studies showed that the positive 222Q/K Greek goats came from heavily infected flocks and that the molecular features of the PrP^res^ were slightly different from the other Greek and Italian scrapie isolates, implying that a different strain may have been present [12]. However, the moderate percentage of the 222K PrP^res^ variant we observed may well indicate that 222K is not a preferential target of this strain.

Strain diversity needs to be taken into account when considering genetic resistance. Because different TSE agents have been reported in sheep and goats [27–29], it is conceivable that different scrapie strains may alter the degree of resistance conferred by the 222K PrP variant. However, recent studies investigating the role of the 222K PrP variant in the susceptibility/resistance of goats to different TSE agents reported that transgenic mice expressing the 222K PrP variant are resistant to a broad panel of goat scrapie isolates and also to cattle BSE agents [6].

In conclusion, our observation of low levels of the 222K PrP variant in the PrP^res^ material of the heterozygous scrapie positive goats corroborates previous findings that, like ARR in sheep, this variant is less prone to conversion into its pathological isoform during prion infection. Our results provide additional evidence that reinforces the view that the 222K *PRNP* allele has a strong protective role against classical scrapie, making it a valid candidate for selective breeding programmes to control this TSE in goats.

## Supplementary Data

19 Supplementary File 1
