# Supplementary material for: Low fraction of the 222K PrP variant in the protease-resistant moiety of PrPres in heterozygous scrapie positive goats
Source: J Gen Virol. 2017 Jul 13;98(7):1963–7. doi: 10.1099/jgv.0.000843 (PMC5656779; doi:10.1099/jgv.0.000843)
Supplement: Supplementary File 1 [file jgv-98-1963-s001.pdf]

Table 1. Raw data of the positive and negative scrapie goats analyzed.

Sample n.	Sample id	PrP 222	Scrapie	Replicate	O.D. F99	O.D. SAF84	ratio F99/SAF84	ratio mean	ratio sd	ratio median	outlier
1	GR 195	Q/K	negative	1	5,2060	16,6140	0,3134	0,3217	0,0083	0,3217	NO
1	GR 195	Q/K	negative	2	25,4270	77,0460	0,3300	0,3217	0,0083	0,3217	NO
1	GR 195	Q/K	negative	3	15,3165	47,6131	0,3217	0,3217	0,0083	0,3217	NO
2	GR 247	Q/K	negative	1	23,3470	106,5480	0,2191	0,2556	0,0365	0,2556	NO
2	GR 247	Q/K	negative	2	14,8030	50,6650	0,2922	0,2556	0,0365	0,2556	NO
2	GR 247	Q/K	negative	3	19,0750	74,6143	0,2556	0,2556	0,0365	0,2556	NO
3	IT 19434	Q/K	negative	1	18,5610	75,9080	0,2445	0,3366	0,0850	0,3532	NO
3	IT 19434	Q/K	negative	2	70,2030	170,3650	0,4121	0,3366	0,0850	0,3532	NO
3	IT 19434	Q/K	negative	3	37,2830	105,5710	0,3532	0,3366	0,0850	0,3532	NO
4	IT 17778	Q/K	negative	1	31,7230	106,4040	0,2981	0,2867	0,0327	0,2981	NO
4	IT 17778	Q/K	negative	2	53,8320	172,4830	0,3121	0,2867	0,0327	0,2981	NO
4	IT 17778	Q/K	negative	3	11,1110	44,4880	0,2498	0,2867	0,0327	0,2981	NO
5	IT 19564	Q/K	negative	1	39,4450	97,0280	0,4065	0,3758	0,0421	0,3930	NO
5	IT 19564	Q/K	negative	2	42,0210	106,9150	0,3930	0,3758	0,0421	0,3930	NO
5	IT 19564	Q/K	negative	3	62,2490	189,8720	0,3278	0,3758	0,0421	0,3930	NO
6	IT 19431	Q/K	negative	1	64,6750	141,0170	0,4586	0,4146	0,0440	0,4146	NO
6	IT 19431	Q/K	negative	2	71,8625	173,3219	0,4146	0,4146	0,0440	0,4146	NO
6	IT 19431	Q/K	negative	3	79,0500	213,3000	0,3706	0,4146	0,0440	0,4146	NO
7	FR 60120	Q/K	negative	1	50,7520	105,2110	0,4824	0,4252	0,0979	0,4809	NO
7	FR 60120	Q/K	negative	2	32,7550	104,9330	0,3122	0,4252	0,0979	0,4809	NO
7	FR 60120	Q/K	negative	3	47,8240	99,4400	0,4809	0,4252	0,0979	0,4809	NO
8	FR 60101	Q/K	negative	1	69,6040	156,6790	0,4442	0,4275	0,0262	0,4409	NO
8	FR 60101	Q/K	negative	2	49,6500	112,6060	0,4409	0,4275	0,0262	0,4409	NO
8	FR 60101	Q/K	negative	3	43,1570	108,6100	0,3974	0,4275	0,0262	0,4409	NO
9	IT 1	Q/Q	negative	1	89,8960	86,1560	1,0434	0,8481	0,1696	0,7639	NO
9	IT 1	Q/Q	negative	2	95,6410	129,7490	0,7371	0,8481	0,1696	0,7639	NO
9	IT 1	Q/Q	negative	3	154,6200	202,4200	0,7639	0,8481	0,1696	0,7639	NO
10	IT 2	Q/Q	negative	1	231,2970	263,8140	0,8767	0,8147	0,0665	0,8229	NO
10	IT 2	Q/Q	negative	2	105,1210	127,7390	0,8229	0,8147	0,0665	0,8229	NO
10	IT 2	Q/Q	negative	3	114,6560	154,0200	0,7444	0,8147	0,0665	0,8229	NO
11	IT 3	Q/Q	negative	1	32,6870	33,1230	0,9868	0,8340	0,2161	0,8340	NO
11	IT 3	Q/Q	negative	2	65,0910	155,5810	0,4184				YES (removed)
11	IT 3	Q/Q	negative	3	92,3270	135,5240	0,6813	0,8340	0,2161	0,8340	NO
12	IT 4	Q/Q	negative	1	106,8500	114,9000	0,9299	0,9478	0,0188	0,9462	NO
12	IT 4	Q/Q	negative	2	148,8340	157,2900	0,9462	0,9478	0,0188	0,9462	NO
12	IT 4	Q/Q	negative	3	270,6910	279,8270	0,9674	0,9478	0,0188	0,9462	NO
13	GR 05	Q/K	positive	1	117,6840	212,5190	0,5538	0,5905	0,2054	0,5169	NO
13	GR 05	Q/K	positive	2	40,2610	83,8680	0,4801	0,5905	0,2054	0,5169	NO
13	GR 05	Q/K	positive	3	20,4790	23,0080	0,8901	0,5905	0,2054	0,5169	NO
13	GR 05	Q/K	positive	4	11,8150	26,9680	0,4381	0,5905	0,2054	0,5169	NO
14	FR 60121	Q/K	positive	1	100,0000	145,1890	0,6888	0,6926	0,0038	0,6926	NO
14	FR 60121	Q/K	positive	2	100,0000	143,6050	0,6964	0,6926	0,0038	0,6926	NO
14	FR 60121	Q/K	positive	3	100,0000	144,3927	0,6926	0,6926	0,0038	0,6926	NO
15	FR 60135	Q/K	positive	1	191,5370	254,7750	0,7518	0,7687	0,0689	0,7518	NO

15	FR 60135	Q/K	positive	2	209,8520	248,4860	0,8445	0,7687	0,0689	0,7518	NO
15	FR 60135	Q/K	positive	3	261,5940	368,5340	0,7098	0,7687	0,0689	0,7518	NO
Sample n.	Sample id	PrP 222	Scrapie	Replicate	O.D. F99	O.D. SAF84	ratio F99/SAF84	ratio mean	ratio sd	ratio median	outlier
16	GR 91	Q/Q	positive	1	58,7030	132,7500	0,4422	0,6465	0,2171	0,5963	NO
16	GR 91	Q/Q	positive	2	183,0680	323,4500	0,5660	0,6465	0,2171	0,5963	NO
16	GR 91	Q/Q	positive	3	97,4450	155,5090	0,6266	0,6465	0,2171	0,5963	NO
16	GR 91	Q/Q	positive	4	72,5800	76,3150	0,9511	0,6465	0,2171	0,5963	NO
17	GR 55	Q/Q	positive	1	122,1510	130,5320	0,9358	0,7625	0,1480	0,7618	NO
17	GR 55	Q/Q	positive	2	183,4100	259,8060	0,7059	0,7625	0,1480	0,7618	NO
17	GR 55	Q/Q	positive	3	89,9090	152,1740	0,5908	0,7625	0,1480	0,7618	NO
17	GR 55	Q/Q	positive	4	259,3950	317,2590	0,8176	0,7625	0,1480	0,7618	NO
18	GR 18	Q/Q	positive	1	89,5820	103,0110	0,8696	0,7116	0,1537	0,7377	NO
18	GR 18	Q/Q	positive	2	60,0260	119,7550	0,5012	0,7116	0,1537	0,7377	NO
18	GR 18	Q/Q	positive	3	188,1230	259,1720	0,7259	0,7116	0,1537	0,7377	NO
18	GR 18	Q/Q	positive	4	196,2670	261,8270	0,7496	0,7116	0,1537	0,7377	NO
19	IT 54817	Q/Q	positive	1	69,8800	78,0880	0,8949	0,7958	0,0902	0,7740	NO
19	IT 54817	Q/Q	positive	2	45,7540	63,6910	0,7184	0,7958	0,0902	0,7740	NO
19	IT 54817	Q/Q	positive	3	97,6690	126,1820	0,7740	0,7958	0,0902	0,7740	NO
20	IT 72366	Q/Q	positive	1	16,7210	23,2740	0,7184	0,7065	0,0778	0,6881	NO
20	IT 72366	Q/Q	positive	2	36,0370	56,4560	0,6383	0,7065	0,0778	0,6881	NO
20	IT 72366	Q/Q	positive	3	16,4760	20,3070	0,8113	0,7065	0,0778	0,6881	NO
20	IT 72366	Q/Q	positive	4	33,6120	51,0990	0,6578	0,7065	0,0778	0,6881	NO
21	IT 77811	Q/Q	positive	1	98,2380	126,4200	0,7771	0,8100	0,0838	0,7771	NO
21	IT 77811	Q/Q	positive	2	88,7600	118,7290	0,7476	0,8100	0,0838	0,7771	NO
21	IT 77811	Q/Q	positive	3	56,6390	62,5710	0,9052	0,8100	0,0838	0,7771	NO
22	IT 7395	Q/Q	positive	1	100,0000	161,6270	0,6187	0,7384	0,1334	0,7249	NO
22	IT 7395	Q/Q	positive	2	100,0000	137,3450	0,7281	0,7384	0,1334	0,7249	NO
22	IT 7395	Q/Q	positive	3	100,0000	144,5540	0,6918	0,7384	0,1334	0,7249	NO
22	IT 7395	Q/Q	positive	4	175,9560	205,1190	0,8578	0,7384	0,1334	0,7249	NO
22	IT 7395	Q/Q	positive	5	100,0000	137,9510	0,7249	0,7384	0,1334	0,7249	NO
22	IT 7395	Q/Q	positive	6	100,0000	171,7170	0,5824	0,7384	0,1334	0,7249	NO
22	IT 7395	Q/Q	positive	7	100,0000	103,6330	0,9649	0,7384	0,1334	0,7249	NO
23	FR 70115	K/K*	negative	1	0,0000	61,4040	0,0000	0,0000	0,0000	0,0000	NO
23	FR 70115	K/K*	negative	2	0,0000	29,2550	0,0000	0,0000	0,0000	0,0000	NO
23	FR 70115	K/K*	negative	3	0,0000	100,0000	0,0000	0,0000	0,0000	0,0000	NO
24	FR 70505	K/K*	positive	1	0,0000	271,2850	0,0000	0,0000	0,0000	0,0000	NO
24	FR 70505	K/K*	positive	2	0,0000	203,2630	0,0000	0,0000	0,0000	0,0000	NO
24	FR 70505	K/K*	positive	3	0,0000	245,0370	0,0000	0,0000	0,0000	0,0000	NO
25	FR 60572	K/K*	positive	1	0,0000	373,3840	0,0000	0,0000	0,0000	0,0000	NO
25	FR 60572	K/K*	positive	2	0,0000	260,3510	0,0000	0,0000	0,0000	0,0000	NO
25	FR 60572	K/K*	positive	3	0,0000	253,6860	0,0000	0,0000	0,0000	0,0000	NO
26	FR 60576	K/K*	positive	1	0,0000	176,0370	0,0000	0,0000	0,0000	0,0000	NO
26	FR 60576	K/K*	positive	2	0,0000	191,2610	0,0000	0,0000	0,0000	0,0000	NO
26	FR 60576	K/K*	positive	3	0,0000	303,7090	0,0000	0,0000	0,0000	0,0000	NO

Gr, Fr, It: Greek, French and Italian goats; * : experimental i.c. scrapie cases; O.D.: optical density; sd : standard deviation.
